# Reproductive Tract Infections (RTI) among married women in Sri Lanka

**DOI:** 10.1186/1742-4690-9-S1-P45

**Published:** 2012-05-25

**Authors:** Sathyadevi Herath, Pushpa Fonseka, Sujatha Samarkoon

**Affiliations:** 1 National Sexually Transmitted Diseases, AIDS Control Program, Ministry Of Health, Sri Lanka; 2 University of Sri Jayawardenepura, Sri Lanka

## Introduction

Feminization of HIV epidemic, and increasing in HIV infection among house wives is seen in most of the world. Yet, community prevalence data on RTI are sparse in Sri Lanka, and little is known about rates among community women. The objective was to describe the prevalence and risk factors of RTI in women aged 22–49years, living in highly populated poor urban settlements of Sri Lanka.

## Methodology

A community-based cross-sectional study was conducted among 770 married women living in 116 urban slums of Colombo city, Sri Lanka. Participants were interviewed on sociodemographic data, sexual history, knowledge on STI/HIV, and condom use. Laboratory specimens were collected for thediagnosis of RTI.

Prevalence was calculated with corresponding 95% confidence intervals (CI). Analyses of risk factors were carried outseparately for the outcomes of sexually transmitted infections: chlamydia, gonorrhoea, trichomoniasis; and endogenous infections:bacterial vaginosis (BV) and candida.

## Results

Ninety three percent of women had single life time partner, only 9% was previously screened for STI/HIV. Condom use was mainly decided by the male partner and none of them used condom for prevention of STI/HIV.

Endogenous infections were relatively common [BV 8.6% (95% CI: 6.6.-10.6)candida 6.8 % ( 95%CI: 5.0-8.6)], and sexually transmitted infections (STI) were infrequent (1.1%-95% CI: 0.34-1.86).

Of the risk factors investigated none of the factors were associated with RTI on multivariate analysis.

## Conclusion

Married women in this community had a low prevalence of RTI and risky sexual behavoiurs were infrequent. Most of the population burden of RTI is attributedto endogenous infections. However, education and outreach screening facilities are needed to reduce the stigma, embarrassment and lack of knowledge related to STI/HIV in order to facilitate screening and condom use.

